# UNDERSTANDING THE INTERPLAY OF BRAIN HEALTH, COGNITION, AND AUTONOMIC FUNCTION

**DOI:** 10.1093/geroni/igad104.3135

**Published:** 2023-12-21

**Authors:** Evan Simms, Sarah Wilson, Roger Newman-Norlund, Julius Fridriksson, Sarah Newman-Norlund

**Affiliations:** Claflin University, Orangeburg, South Carolina, United States; University of South Carolina, Columbia, South Carolina, United States; University of South Carolina, Columbia, South Carolina, United States; University of South Carolina, Columbia, South Carolina, United States; University of South Carolina, Columbia, South Carolina, United States

## Abstract

Research in clinical populations focused on various age-related diseases report multi-system difficulties in autonomic regulation, motor performance, cognition, and brain health. My research investigated the relationship between brain health, chronological age, and cognition. To assess these domains, I cross analyzed Compass 31, MoCA, and Brain Age Technology. The study population included 158 participants, both healthy and unhealthy. The collected data evaluated the relationship between brain age disparity, chronological age, and autonomic function. Correlation analysis, regression modeling, and subgroup comparisons were employed to explore potential associations, age-related patterns, and autonomic function variations. The findings from this research study contributes to a deeper understanding of the complex interplay between brain health, chronological age, and autonomic function. Age, White Matter Hyperintensities, Brain Age Gap, and Brain Age Variance demonstrate high significance when correlated with the MoCA total score. MoCA and all Compass 31 domains were found to be predictive of total white matter hyperintensity volume. This finding highlights a strong relationship between all three domains and suggests that when participants score high on both assessments, they should follow up with neuroimaging to discover potential brain damage. Future studies should identify risk factors for cognitive impairment and brain health that are also symptoms for autonomic dysregulation.

